# The effects of robot-assisted laparoscopic surgery with Trendelenburg position on short-term postoperative respiratory diaphragmatic function

**DOI:** 10.1186/s12871-024-02463-3

**Published:** 2024-03-05

**Authors:** Shuo Xue, Dan Wang, Hong-Qin Tu, Xiao-Ping Gu, Zheng-Liang Ma, Yue Liu, Wei Zhang

**Affiliations:** Department of Anesthesiology, Nanjing Drum Tower Hospital, The Affiliated Hospital of Nanjing University Medical School, No.321 of Zhongshan Road, Nanjing, 210008 Jiangsu Province China

**Keywords:** Robot-assisted laparoscopic surgery, Diaphragm ultrasound, Lung injury, Airway pressure, Lung compliance

## Abstract

**Objective:**

To study how Pneumoperitoneum under Trendelenburg position for robot-assisted laparoscopic surgery impact the perioperative respiratory parameters, diagrammatic function, etc.

**Methods:**

Patients undergoing robot-assisted laparoscopic surgery in the Trendelenburg position and patients undergoing general surgery in the supine position were selected. The subjects were divided into two groups according to the type of surgery: robot-assisted surgery group and general surgery group. ① Respiratory parameters such as lung compliance, oxygenation index, and airway pressure were recorded at 5 min after intubation, 1 and 2 h after pneumoperitoneum. ② Diaphragm excursion (DE) and diaphragm thickening fraction (DTF) were recorded before entering the operating room (T_1_), immediately after extubation (T_2_), 10 min after extubation (T_3_), and upon leaving the postanesthesia care unit (T_4_). ③ Peripheral venous blood (5 ml) was collected before surgery and 30 min after extubation and was analyzed by enzyme-linked immunosorbent assay to determine the serum concentration of Clara cell secretory protein 16 (CC16) and surfactant protein D (SP-D).

**Result:**

① Compared with the general surgery group (*N* = 42), the robot-assisted surgery group (*N* = 46) presented a significantly higher airway pressure and lower lung compliance during the surgery(*P* < 0.001). ② In the robot-assisted surgery group, the DE significantly decreased after surgery (*P* < 0.001), which persisted until patients were discharged from the PACU (*P* < 0.001), whereas the DTF only showed a transient decrease postoperatively (*P* < 0.001) and returned to its preoperative levels at discharge (*P* = 0.115). In the general surgery group, the DE showed a transient decrease after surgery(*P* = 0.011) which recovered to the preoperative levels at discharge (*P* = 1). No significant difference in the DTF was observed among T_1_, T_2_, T_3_, and T_4_. ③ Both the general and robot-assisted surgery reduced the postoperative serum levels of SP-D (*P* < 0.05), while the robot-assisted surgery increased the postoperative levels of CC16 (*P* < 0.001).

**Conclusion:**

Robot-assisted laparoscopic surgery significantly impairs postoperative diaphragm function, which does not recover to preoperative levels at PACU discharge. Elevated levels of serum CC16 after surgery suggest potential lung injury. The adverse effects may be attributed to the prolonged Trendelenburg position and pneumoperitoneum during laparoscopic surgery.

## Introduction

Robot-assisted laparoscopic surgery was evolved to expand the patient base being offered a minimally invasive approach while maximally overcoming the associated limitations of minimally invasive surgery. Due to the tremendous value the robot adds to surgery, it has been widely used in various surgical specialties, however, attendant complications are not rare. The unique logistics of robot-assisted surgical configurations, particularly specialized patient positioning coupled with prolonged surgical times, are key risk factors for pulmonary complications [1, 2].

The Trendelenburg position, where the patient’s head is lowered and the feet are raised above the level of the head, is commonly performed in various surgical procedures, especially in robot-assisted surgeries such as robotic lower abdominal surgery, robot-assisted genitourinary surgery, and robot-assisted gynecologic surgery [3]. With the patient in the Trendelenburg position, gravity forces to keep the intestines away from the pelvis and abdominal cavity, which improves visualization and accessibility by creating more space in the surgical area and reduces the risk of injury by avoiding unexpected movement of organs and tissues. Although the Trendelenburg position provides numerous benefits during robot-assisted surgery, it is not without potential dangers, with the lungs and diaphragm bearing the brunt [4, 5]. On one hand, the displacement of abdominal organs towards the chest leads to decreased lung volumes and compliance. On the other hand, increased intrathoracic pressure and altered abdominal mechanics reduce the diaphragm’s excursion and even induce paradoxical motion thereby limiting the formation of negative pressure. All of the above may result in impaired gas exchange and increased work of breathing, which will be further exacerbated by pneumoperitoneum during laparoscopic surgery [6]. Therefore, special considerations and strategies to closely monitor patients’ respiratory physiology and mitigate these adverse impacts are extremely important for healthcare professionals.

Diaphragm ultrasound has been validated since 1975, while it has only recently gained ground in the field of anesthesia owing to studies indicating a strong link among failure of mechanical weaning, postoperative pulmonary complications, prolonged hospital stay and diaphragmatic dysfunctions [7–9]. These studies have also raised interest in the use of diaphragm ultrasound as a reference tool for predicting prognosis in surgical patients. As a new window available to obtain information about dynamics of the thoracic-pulmonary system, we used diaphragm ultrasound to assess the respiratory workload, focusing on the impact of robot-assisted laparoscopic surgery on early postoperative respiratory function in patients with general anesthesia. Meanwhile, we further determine lung injury by monitoring changes in intraoperative respiratory parameters and serum concentrations of lung injury markers including Clara cell secretory protein 16 (CC16) and surfactant protein D (SP-D).

## Method

### Inclusion and exclusion criteria

Inclusion criteria: (1) Patients undergoing robot-assisted surgery requiring Trendelenburg position and pneumoperitoneum in the anesthesia and operating room of Nanjing Drum Tower Hospital between January 2022 and December 2022, with the main type of surgery being robot-assisted laparoscopic radical prostate/bladder cancer; During the operation, The patient was then placed in a 15°Trendelenburg position, and pneumoperitoneum was maintained at 12 mmHg. Trocar configurations include 2-cm above the umbilicus, ateral margin of rectus abdominis, and anterior axillary line, etc. Surgical steps of robot-assisted laparoscopic radical prostatectomy involve creating a vertical cystotomy, dissecting intravesical structures, performing nerve-sparing dissection, and suturing the urethrovesical anastomosis, etc [10];(2) Patients undergoing general surgery without pneumoperitoneum and in a horizontal position in the anesthesia and operating room of Nanjing Drum Tower Hospital from January 2022 to December 2022, with the main type of surgery being total knee arthroplasty. Surgical steps of total knee arthroplasty involve performing a lateral parapatellar arthrotomy, releasing the Hoffa fat pad, performing bony cuts, and implanting artificial prosthesis, etc [11]. Age range: 18 to 80 years. ASA physical status classification: ASA I to III. Ethical approval obtained from the ethics committee of Nanjing Drum Tower Hospital with approval number: 2021-499-01.

Exclusion criteria: (1) lung disease; (2) history of thoracic and abdominal surgery; (3) diaphragmatic dysfunction; (4) thoracic deformity; (5) body mass index (BMI) ≥ 30 kg/m^2^; (5) neuromuscular disease; (6) poor quality of ultrasound window; (7) abnormal lung function; (8) severe cardiac disease; (9) coexisting lung infection.

Sample size: Pre-experiment results indicate that the difference in DE before and after surgery is 7.1 ± 3.8 mm in the robot-assisted surgery group, and the average difference in DE before and after surgery is 4.7 ± 4.5 mm in the general surgery group. Using α = 0.05, and power = 0.8, the calculated paired sample size is *N* = 36. Considering a dropout rate of 10%, the total sample size for both groups is 80 cases.

### Data collection

Observation Parameters: The study includes respiratory parameters, perioperative parameters, diaphragm ultrasound parameters, and lung injury parameters. Diaphragm ultrasound and lung injury parameters are the main study parameters, while respiratory parameters, perioperative parameters, and general subject information are secondary parameters. The details and measurement methods of the observation indexes are as follows.

Respiratory parameters: Tidal volume, oxygenation index, partial pressure of end-tidal carbon dioxide (PetCO_2_), dynamic lung compliance (C_dyn_), and peak airway pressure (P_peak_) were recorded at 5 min after intubation, 1 and 2 h after pneumoperitoneum.

Perioperative parameters: heart rate, mean artery pressure (MAP), central venous pressure (CVP), and other circulatory parameters were recorded 5 min after intubation, 1 and 2 h after pneumoperitoneum. At the end of the operation, intraoperative fluid, bleeding, and urine volume were recorded; at the time of extubation, the duration of mechanical ventilation and the level of visual analogue scale (VAS) of pain were recorded.

**Fig. 1:**
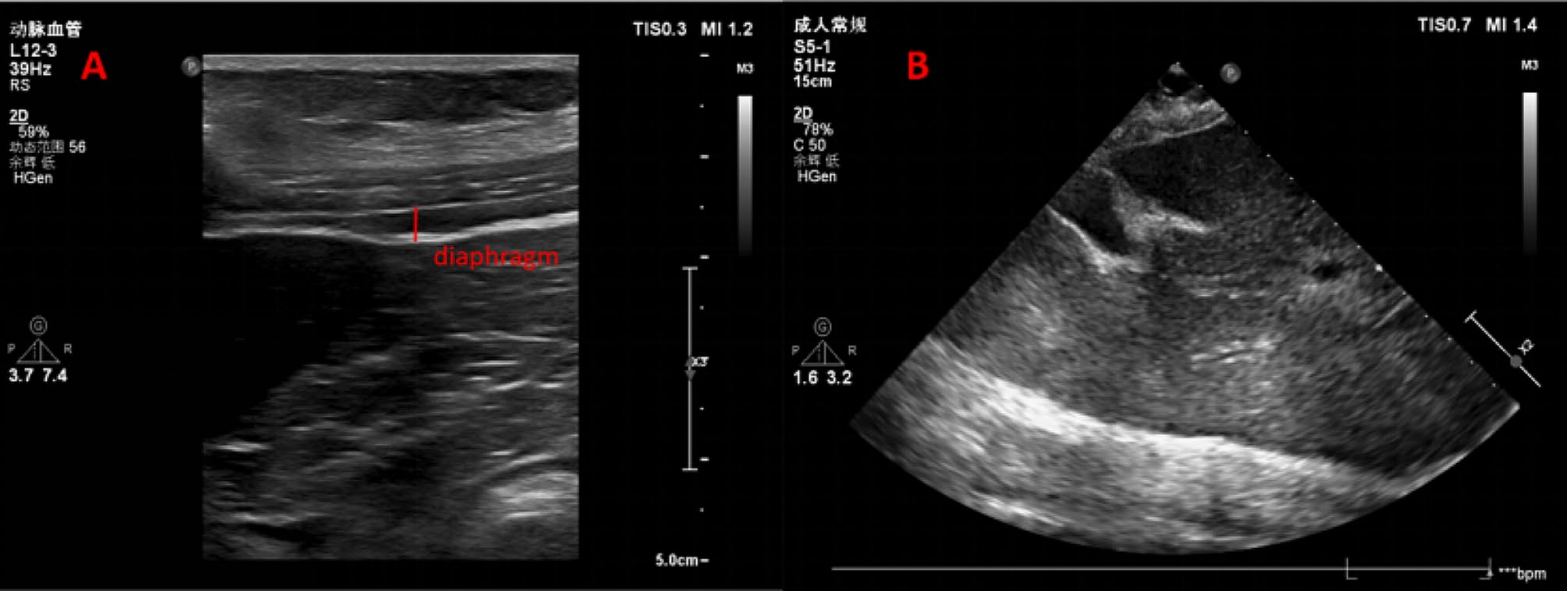
Diaphragm ultrasound images. **A** DTF measured by high-frequency probe in horizontal position; **B** DE measured by low-frequency probe in semi-recumbent position

Diaphragm ultrasound parameters: (1) Diaphragm thickening fraction (DTF): The subject was placed in horizontal position, and a 10 ∼ 15 MHz line array probe was placed in the 8th to 11th intercostal space between the mid-axillary line and the anterior axillary line on the left side of the subject, and a three-layer structure was visible as the diaphragm at a depth of 2 ∼ 4 cm (Fig. 1A). The diaphragm thickness was measured at the end of expiration and the end of inspiration, and the DTF = (end-inspiratory diaphragm thickness - end-expiratory diaphragm thickness)/ end-expiratory diaphragm thickness * 100%. Each subject measured 3 respiratory cycles to calculate the average value; (2) Diaphragm excursion (DE): the subject was placed in the semi-recumbent position, and a 2 ∼ 5 MHz curved array probe was placed at the mid-clavicular line directly below the left rib arch. The ultrasound beam was made as perpendicular to the diaphragm as possible, and a bright line could be seen covering the surface of the liver, which was the diaphragm (Fig. 1B). The distance traveled by the diaphragm during the respiratory cycle was then recorded vertically by changing the mode from B to M. Each subject measured 3 respiratory cycles to calculate the average value [8, 12]. The diagnostic ultrasound machine model was Philips CX50.

Lung injury parameters: Peripheral venous blood(5 ml) was taken from the subjects before operation and 30 min after extubation, respectively; after centrifugation at 3000r/min for 10 min, the upper layer of the clear liquid was taken, and the levels of serum CC16 and SP-D were detected by enzyme-linked immunosorbent assay(Elisa). Human SP-D Elisa kit was provided by MultiSciences(Lianke) Biotechnology Co., Ltd, Human CC16 Elisa kit was provided by Shanghai Enzyme-linked Biotechnology Co., Ltd, and the enzyme marker was provided by Thermo Fisher Scientific (China) Co.

### Anesthesia procedure

Patients are monitored with electrocardiography, blood pressure, pulse oximetry, bispectral index (BIS), and invasive blood pressure via radial artery catheterization in perioperative phase. The anesthesia regimen was as follows:(1) induction regimen: midazolam 50 μg/kg compound propofol 1 mg/kg or etomidate 0.2-0.3 mg/kg, vancoson 160 μg/kg, and fentanyl 4 ∼ 6 μg/kg; (2) maintenance regimen: propofol 4 ∼ 6 mg/kg/h, atracurium 80 μg/kg/h, remifentanil 0.05–0.1 μg/kg/min; (3) BIS monitoring was maintained between 40 ∼ 60. Various vasoactive drugs currently commonly used in clinical practice could be used selectively during operation according to the hemodynamic alterations: ephedrine, phenylephrine, and atropine. All drugs were administered intravenously. Intraoperative ventilation mode was volume control ventilation, with a tidal volume of 8 ml/kg, oxygen flow rate of 2 L/min, inhaled oxygen concentration of 100%, inspiratory/expiratory ratio of 1:1.5, respiratory rate of 12 breaths/min, and no positive end-expiratory pressure, which could be appropriately adjusted according to the patient’s PetCO_2_ level. At the end of the surgery, neostigmine 40pg/kg and atropine 20 μg/kg were given.

The extubation criteria are as follows: (1) The patient is conscious with a BIS value greater than 80, can open eyes on command, and has restored coughing and swallowing reflexes. (2) The ability to lift the head for more than 5 s, and strong hand raising and fist clenching. (3) Regular and steady breathing, with a rate of 10 ∼ 20 breaths/min, and tidal volume reaching 6 ∼ 7 ml/kg. (4) PetCO_2_ less than or equal to 45mmHg. After extubation, oxygen is provided via a face mask at a flow rate of 5 L/min, and observation continues for more than 30 min. Patients with a Visual Analogue Scale(VAS) of three and higher were given fentanyl 25–50 μg, repeated if necessary. Before each diaphragm ultrasound, subjects have to undergo VAS assessment, and the diaphragm ultrasound is only performed if the score is less than or equal to two. The patient can leave the postanesthesia care unit (PACU) and be transferred to the ward only after being assessed with a Steward score of 6.

### Experimental process

**Fig. 2:**
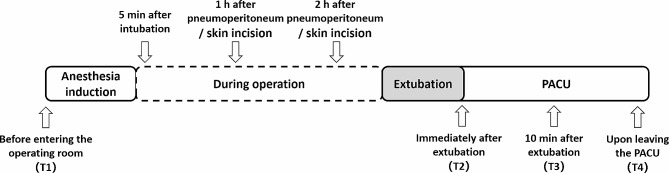
Study protocol. Four predefined time points (T1 ∼ T4) when diaphragm ultrasound were performed and perioperative variables were recorded during the operation

(1) Patients were screened according to the inclusion and exclusion criteria, and divided into robot-assisted surgery and general surgery groups.

(2) The patients’ age, gender, height, BMI, ASA classification, diseases, and other general information were recorded before surgery.

(3) Respiratory parameters such as tidal volume, oxygenation index, PetCO_2_, pulmonary compliance and airway pressure, and circulatory parameters such as heart rate, MAP, and CVP were recorded 5 min after intubation, 1 and 2 h after pneumoperitoneum(robot-assisted surgery group)/skin incision(general surgery group). Diaphragm ultrasound parameters (DE and DTF) are recorded before entering the operating room (T1), immediately after extubation (T2), 10 min after extubation (T3) and upon leaving the PACU (T4); peripheral venous blood(5 ml) was taken from the patients before and 30 min after extubation, and the upper layer of the supernatant was centrifuged at 3,000 r/min for 10 min, then the concentrations of serum CC16 and SP-D were detected using the Elisa (Fig. 2).

### Statistical methods

Data will be analyzed using SPSS Version 26.0. Normal data will be presented as mean ± standard deviation and analyzed using one-way repeated measures ANOVA. Non-normal data will be expressed as medians (25th and 75th percentiles) and analyzed using the Wilcoxon signed-rank and Friedman tests with Bonferroni correction. Between-group comparisons will be made using the Mann-Whitney U test. A significance level of *P* < 0.05 will be considered statistically significant.

## Result

### General information

**Fig. 3:**
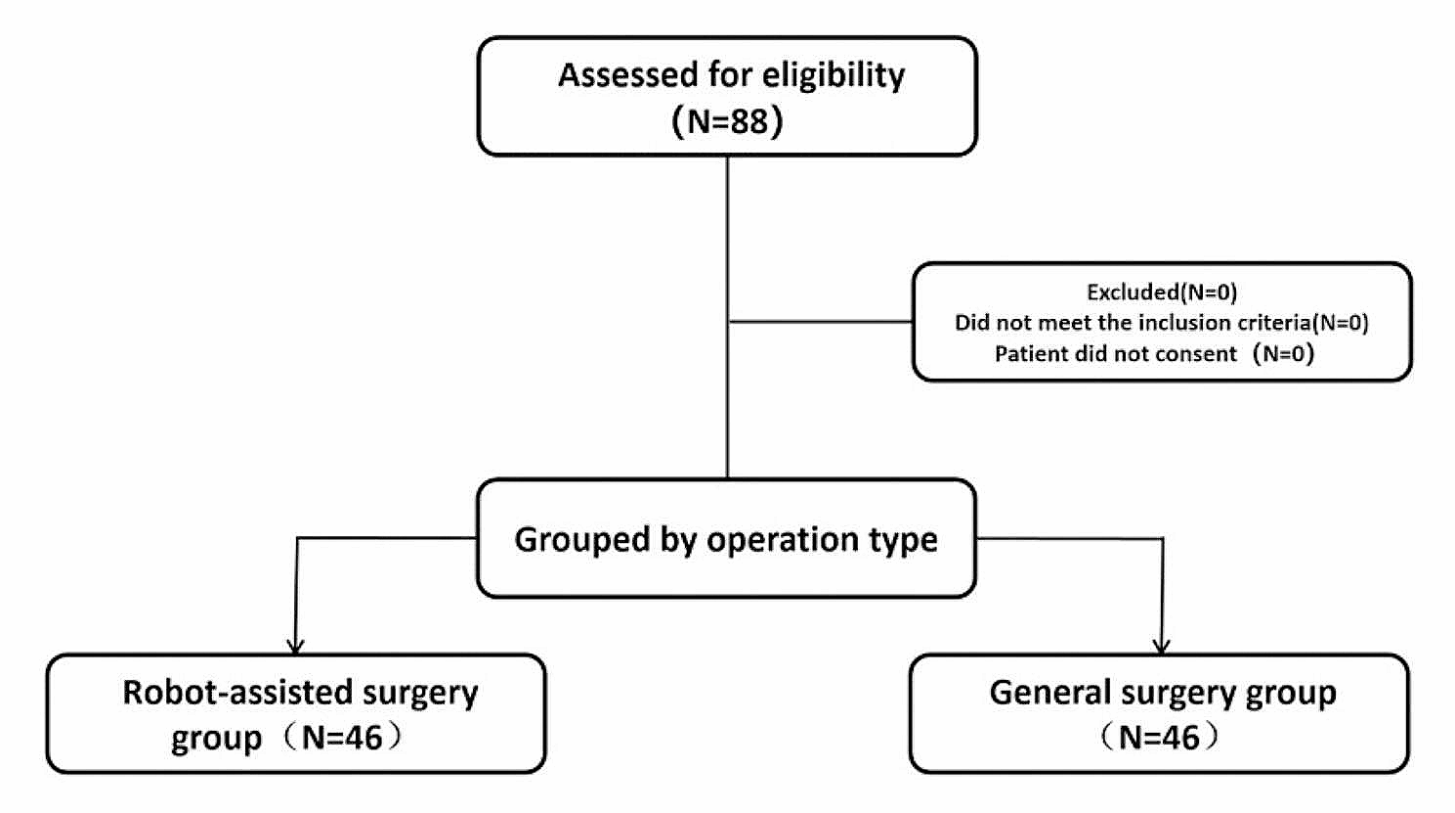
Flow diagram representing patient enrollment and group assignment

A total of 88 participants were enrolled in the study, comprising 46 individuals in the robot-assisted surgery group and 42 in the general surgery group (Fig. 3). There were no statistically significant differences between the two groups concerning demographic information, duration of mechanical ventilation, and intraoperative fluid administration (*P* > 0.05) (Table 1).

**Table 1:** Demographic and perioperative characteristics of the study patients

Variables	robot-assisted surgery group(*N* = 46)	general surgery group(*N* = 42)	*P* value
Age(years)	69(65,74)	66(62,70)	0.136
Body mass index (kg/m^2^)	24.47 ± 2.16	24.93(23.55,27.05)	0.179
ASA physical status(II/III)	16/30	15/27	0.927
Mechanical ventilation duration(min)	195(176.25,230)	190(175,213.75)	0.286
Fluid infusion(ml)	2000(1500,2075)	1600(1500,2000)	0.081
Operative blood loss(ml)	100(100,100)	100(50,200)	0.551
Urine output(ml)	400(200,575)	500(300,787.5)	0.087
Postoperative visual analogue scale	2(1,2)	1(1,2)	0.246
Preoperative CC16(ng/ml)	2.24(0.47, 7.57)	2.87(1.63, 5.78)	0.255
Preoperative SP-D(pg/ml)	246.22(159.81, 471.11)	221.46(170.57, 397.48)	0.382

CC16: Clara cell secretory protein 16, SP-D: surfactant protein D

### Intraoperative changes in respiratory parameters

In the robot-assisted surgery group, a notable increase in peak airway pressure and a significant decrease in lung compliance were observed following pneumoperitoneum initiation (*P* < 0.001). Conversely, these parameters remained relatively stable in the general surgery group. Both groups exhibited a significant rise in the partial pressure of arterial carbon dioxide (PaCO_2_) as the mechanical ventilation time extended (*P* < 0.001). However, no significant differences in the oxygenation index and PaCO_2_ levels were noted between the two groups (*P* = 0.358 and *P* = 0.150, respectively) (Table 2).

**Table 2:** Intraoperative pulmonary variables during the operation

Variables	Group	5 min after intubation	1 h after pneumoperi-toneum/ skin incision	2 h after pneumoperi-toneum/ skin incision	*P* value	
					Group×time	Intergroup
Oxygenation index	robot-assisted surgery group	325.9 ± 96.9	329.6 ± 62.3	329.5 ± 63.7	0.237	0.463
	general surgery group	310.9 ± 132.7	318.6 ± 69.9	297.8 ± 65.7		
PaCO_2_ (mmHg)	robot-assisted surgery group	31.5 ± 3.6	36.9 ± 4.2	37.4 ± 5.7*	<0.001	0.150
	general surgery group	32.7 ± 4.0	33.1 ± 4.3	35.1 ± 4.0		
Peak airway pressure(mmHg)	robot-assisted surgery group	16.8 ± 4.5	25.0 ± 4.0	23.5 ± 4.9*	<0.001	<0.001
	general surgery group	15.3 ± 2.7	16.4 ± 2.6	17.1 ± 2.4		
Lung compliance (ml/mmH_2_O)	robot-assisted surgery group	47.2 ± 14.2	26.7 ± 8.5	28.5 ± 9.7*	<0.001	<0.001
	general surgery group	38.4 ± 6.1	34.9 ± 5.2	34.6 ± 4.9		

PaCO_2_: Partial Pressure of Carbon Dioxide; *: *p*-value < 0.05

### Postoperative diaphragm movement changes

In the robot-assisted surgery group, there were significant variations in Diaphragm Excursion (DE) at times T1 vs. T2, T3, and T4 (*P* < 0.001), as well as between T2 and T4 (*P* < 0.001). For the general surgery group, a significant change in DE was observed only between T1 and T2 (*P* = 0.011). No statistically meaningful differences in DE were found between T1 vs. T3 or T4 (corrected *P* = 1) or between T2 and T4 (*P* = 0.108) (Table 3).

Significant changes in Diaphragm Thickness Fraction (DTF) were found between T1 and T2, as well as between T1 and T3 in the robot-assisted group (*P* < 0.001). No significant difference in DTF was noted between T1 and T4 (*P* = 0.115). A significant divergence between T2-DTF and T4-DTF was observed (*P* = 0.004). In the general surgery group, no statistically significant alterations in DTF were seen across T1, T2, T3, and T4 (*P* > 0.05) (Table 3).

**Fig. 4:**
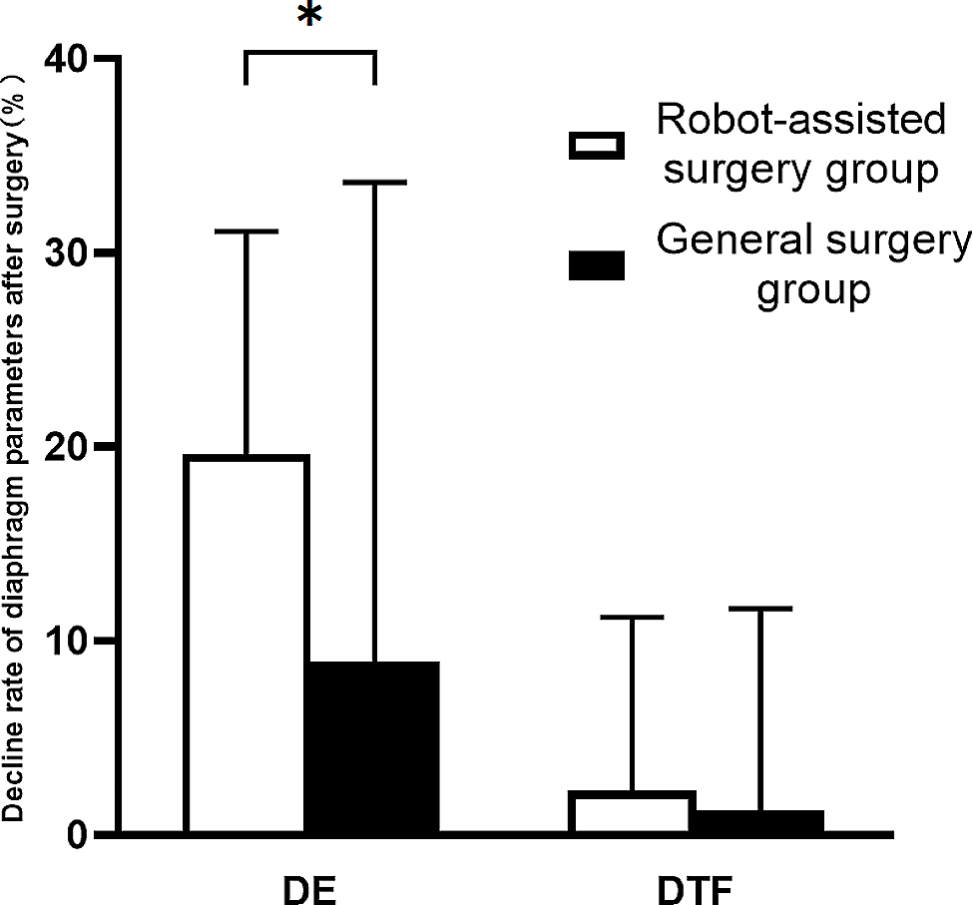
Decline rate of diaphragm parameters after surgery in two groups; Data are presented as mean ± S.D. Statistical signifcance was tested using Mann-Whitney U test in GraphPad Prism (version 8, GraphPad Software, San Diego, CA, USA). *: *p*-value < 0.05

The postoperative reduction in DE was 19.68% (10.83, 26.53%) in the robot-assisted surgery group compared to 1.31% (-1.83, 6.95%) in the general surgery group, indicating a significant difference (*P* < 0.001) (Fig. 4).

**Table 3:** Diaphragm variables at each time point

Variables	Group	T_1_	T_2_	T_3_	T_4_
DE(cm)	robot-assisted surgery group	1.84(1.54, 2.05)	1.25(1.06, 142)**†**	1.29(1.15, 1.60)	1.46(1.19, 1.62)*#
	general surgery group	1.51(1.36, 1.79)	1.48(1.27, 1.70)**†**	1.52(1.32, 1.65)	1.52(1.34, 1.67)
DTF	robot-assisted surgery group	0.42(0.37, 0.45)	0.33(0.27, 0.38)**†**	0.35(0.32, 0.40)	0.35(0.32, 0.40)**#**
	general surgery group	0.38(0.36, 0.46)	0.39(0.35, 0.42)	0.38(0.35, 0.42)	0.40(0.36, 0.42)

DE: diaphragm excursion, DTF: diaphragm thickening fraction. †: *p*-value < 0.05;*: *p*-value < 0.05; #: *p*-value < 0.05

### Postoperative lung injury parameters’ changes

As presented in Table 4, postoperative levels of SP-D significantly decreased in both the robot-assisted and general surgery group compared to preoperative levels (*P* < 0.05). Conversely, postoperative levels of CC16 in the robot-assisted group showed a significant increase compared to their preoperative levels (*P* < 0.001). No such increase was observed in the general surgery group (*P* = 0.083). Furthermore, postoperative CC16 levels in the robot-assisted surgery group were markedly higher than those in the general surgery group (*P* < 0.001) (Table 4).

**Table 4:** Perioperative serum lung injury markers comparison

Variables	Group	Before operation	30 min after extubation	*P* value
SP-D (pg/ml)	robot-assisted surgery group	246.22(159.81, 471.11)	212.43(160.23, 520.77)	0.033
	general surgery group	221.46(170.57, 397.48)	188.31(170.60, 306.92)	<0.001
CC16 (ng/ml)	robot-assisted surgery group	2.88(1.63, 5.79)	4.51(2.47, 8.54)	<0.001
	general surgery group	2.11(0.36, 6.18)	3.29(1.12, 7.23)	0.083

SP-D: Surfactant protein D; CC16: Clara cell secretory protein 16

## Discussion

Robot-assisted laparoscopic surgery imposes unique challenges on patients’ postoperative respiratory function due to prolonged Trendelenburg positioning and pneumoperitoneum. In the present study, we noted significant alterations in respiratory mechanics among patients undergoing robot-assisted procedures, specifically increased airway pressures and diminished lung compliance. These changes coincide with the established high-risk factors for ventilator-associated lung injury (VALI) such as elevated airway pressures and high tidal volumes [13]. We observed notable impairments in diaphragmatic movement after surgery, a failure to return to baseline levels in a short term, and a significant rise in serum markers of lung injury.

The etiology of perioperative lung injury in robot-assisted radical prostatectomy is complex, with contributing factors such as volutrauma, barotrauma, atelectrauma, and biotrauma [14]. Traditional ventilation strategies often employ tidal volumes greater than 10 ml/kg to prevent hypercapnia. High tidal volume can lead to alveolar overexpansion (volutrauma) and sustained high airway pressure in the airway(barotrauma), which are important risk factors for VALI [15]. The effects of patient positioning and pneumoperitoneum on robot-assisted radical prostatectomy patients result in the upward displacement of the diaphragm, reduced lung volume, and sustained high airway pressure, leading to a series of lung tissue injuries such as barotrauma and atelectrauma [16]. Protective lung ventilation strategies, incorporating reduced tidal volumes, higher respiratory rates, and suitable positive end-expiratory pressure (PEEP), can mitigate these risks. Appropriate PEEP ventilation can balance the lateral displacement of the diaphragm, increase lung volume, and reduce the incidence of atelectasis [16, 17]. Studies have shown that a high PEEP level of 15 cm H_2_O during robot-assisted radical prostatectomy can improve ventilation in gravity-dependent areas of the lung, achieving gas exchange in the lung that is closer to physiological conditions [18]. Despite the substantial clinical evidence confirming the benefits of lung protective ventilation strategy in reducing acute lung injury and the incidence of postoperative pulmonary complications (PPCs), the evidence for applying lung protective ventilation strategy in the general perioperative population remains unclear. A study by Santer P et al. has shown a significant correlation between increased respiratory frequency and the occurrence of PCCs [19]. High-frequency ventilation can increase lung tension, intrapulmonary pressure, and dead cavity volume, leading to hypercapnia. Additionally, there is still controversy regarding whether individuals without preoperative lung conditions can benefit from protective lung ventilation strategy [20].

The diaphragmatic function was particularly compromised in the robot-assisted surgery group, with significant decreases in DE and DTF. DE refers to the distance the diaphragm moves from end-expiration to end-inspiration in a single breath, while DTF is the ratio of diaphragm thickening from end-expiration to end-inspiration. Both DE and DTF are closely related to patient lung function and inspiratory effort [21, 22]. In this study, patients undergoing robot-assisted surgery exhibited a significant decrease in DE during extubation compared to patients undergoing general surgery. While some recovery was noted in the post-anesthesia care unit (PACU), levels failed to return to baseline before discharge. Patients undergoing robot-assisted surgery also experienced a transient decrease in DTF after surgery, while returned to baseline before discharge. The Trendelenburg position and pneumoperitoneum force the diaphragm towards the cranial side, leading to a decrease in lung volume and an increase in airway pressure. It has been suggested that the decrease in lung volume during surgery and the anatomical characteristics of the diaphragm itself are possible reasons for postoperative diaphragm movement impairment, although this view remains contentious [5]. Factors contributing to diaphragmatic dysfunction are multifaceted. Previous studies have shown that prolonged mechanical ventilation can lead to significant atrophy of diaphragm muscle fibers, and control mode ventilation are significant influencing factor in diaphragm dysfunction [23, 24]. Moreover, compared to general surgery, the robot-assisted laparoscopic surgery requires higher muscle relaxation, and the traditional extubation process dominated by muscle strength, consciousness, and spontaneous breathing has been shown to potentially lead to the development of muscle relaxation residuals [25]. Residual muscle relaxation is also one of the possible reasons for impaired diaphragm function after surgery. As the patient’s diaphragm is in a myorelaxed state during surgery and diaphragmatic movement is generated by pressure-driven generation from the ventilator, changes in DE and DTF during mechanical ventilation were not observed in this study.

Our study also noted significant changes in serum markers of lung injury, specifically surfactant protein D (SP-D) and Clara cell secretory protein 16 (CC16). Serum concentrations of SP-D and CC16 are elevated when alveolar-capillary barrier function is impaired. The serum concentrations of CC16 and SP-D have been shown to be strongly related to the severity of lung injury [26, 27].

SP-D is a collagen glycoprotein secreted by type II alveolar cells and Clara cells, participating in lung inflammation and immune response processes [28]. Elevated serum SP-D concentrations have been proven to be closely related to pneumonia, chronic obstructive pulmonary disease, and PPCs [29]. Changes in serum SP-D concentrations during the perioperative period are related to elevated alveolar permeability. Alveoli with decreased compliance repeatedly collapse and re-expand during the respiratory cycle, and high-intensity shear forces on the alveolar surface lead to sustained damage to alveolar epithelial cells and vascular endothelial cells. Previous studies have shown that serum SP-D concentrations remain unchanged after elective surgery, whereas patients undergoing cardiac surgery had significantly higher postoperative SP-D compared to preoperative levels [30, 31]. In this study, the postoperative SP-D levels of both groups were significantly lower than their preoperative levels, which is consistent with the results of Serpa Neto et al. [32]. With its high relative molecular mass (560 kDa), mild lung injury and elevated alveolar permeability did not result in a significant increase in serum SP-D concentrations. The dilution of serum SP-D concentrations by positive perioperative fluid balance is the possible cause of the postoperative decrease in SP-D concentrations.

CC16 is a small protein (16 kDa) secreted by Clara cells in the respiratory epithelium. When the alveolar-capillary membrane barrier is compromised, CC16, which is mainly present in the alveolar epithelial lining fluid, enters the bloodstream in large quantities [26]. Compared to SP-D, CC16 has a lower relative molecular mass, making it easier to pass through the alveolar-capillary membrane barrier. In this study, the postoperative CC16 levels of patients in the robot-assisted surgery group were significantly higher than their preoperative levels, while there were no statistically significant differences in CC16 levels before and after surgery in the general surgery group. The postoperative CC16 levels in the robot-assisted surgery group were significantly higher than those in the general surgery group. Alveolar hyperexpansion due to high airway pressures during surgery in the robot-assisted surgery group was the likely cause of the elevated postoperative CC16 levels compared with patients in the general surgery group. However, these observations are confined to the immediate PACU period, representing a study limitation.

## Conclusion

In summary, our findings indicate that patients undergoing robot-assisted laparoscopic surgery experience significant postoperative impairments in diaphragmatic function, which do not return to baseline levels at PACU discharge. Elevated postoperative serum CC16 levels were also notable, suggesting that the prolonged Trendelenburg position and pneumoperitoneum might be contributing factors. The study’s limitations, including its single-center focus and limited follow-up period, necessitate further research to corroborate these findings.

## Data Availability

The data are not publicly available due to our laboratory’s confidentiality policy. If editors, reviewers, or other researchers want to request the data from this study, please contact Dr Xue(xueshuo23@163.com).
